# The Paris Exhibition

**Published:** 1856-01

**Authors:** 


### ARTICLE VI.

### *The Paris Exhibition.*

Those of our readers who have not had the opportunity or inclination to visit this exhibition of European industry, will, like ourselves, be disappointed when we state, that with but few exceptions, there was scarcely an article exhibited belonging to the dental department, which could consistently be termed a "novelty" in dental science. In truth, we preambulated over and over again this huge building to discover the whereabouts of the dental instrument maker, but without success; it is true, we chanced to alight upon a *surgeon's* instrument maker's stall, which contained circular jointed forceps. The instruments or tools found at the other stalls, were of the most antediluvian character, consisting of forceps, the old fashioned key and punches. We could nowhere discover the splendid exhibition of dental instruments by Chevalier or Arnold, of America, or Dixon & Everard, of London. In fact, we almost began to doubt whether the exhibition contained things dental, had we not been attracted by a huge imitation of a set of carved ivories, and a profusion of ornamental address cards and *professional* circulars. As a matter of course, we were prepared to expect the "Gallic Cock," to have been upon a par at least with England and America in dental science, and we had also been led to suppose, from what has been said and written upon the subject, that in some things they might have stolen a march, particularly in one branch, mechanical dentistry; these impressions, however, were soon obliterated upon inspection. We opine, neither American or English dental practitioners have nothing to fear from the contents of the French exhibition, as far as regards inventions, style, finish or solidity of workmanship ; we are, moreover, inclined to think there is a vast deal more theory than practice, more shadow than substance, a common failing with the majority of the dental profession in all countries. There were but very few exhibitors from either America or England, and a marked difference in the character and importance in the contents, when compared with the Londom exhibition; there was a perceptible want of Chevalier & Arnold's dental instrument stall, also Weiss, Everard, &c., of London. The contributions of these gentlemen to the London exhibition, formed a striking feature, not easily forgotten by those who really take an interest in their profession. The reason probably these exhibitors, and many others, did not contribute to the Paris exhibition arose from the gross neglect and breach of faith practiced towards them by the "council" of the London exhibition, who, in their voluminous report upon the contents of the exhibition generally, made no special mention of anything appertaining to dental science. This alone, we should say, was sufficient to account for the "barren" show by foreigners at the great Paris Bazaar.

We cannot conclude these remarks without according to Drs. Evans, Delabarre, Tucker, Page, &c., our most cordial and hearty thanks for the kind attention and politeness we received at their hands, there was a total absence of "*starch*" affectation and presumption; there was not a particle of the bombast and egotistical conceit of the dental Mæcenas of Wimbledon; there was, as regards those gentlemen, indeed, a most *perfect* "absence of affectation or mannerism." They are, at least, modest, and retiring, and can well afford to let *others* find out whatever merit they possess.

*Contents.—America.*—No. 45. Messrs. Jones, White & McCurdy.—This firm exhibited at the London exhibition, and on that occasion we remarked upon the want of *tube* teeth for the "English market," combining their present exquisite shape and color. But Messrs. Jones, White & Co., are staid "old fellows," and have a mortal antipathy to any kind of innovation. Their exhibition case contains an immense variety of plate teeth, gum teeth and pivoted teeth, all of excellent quality, they are certainly very happy, in shape and color, in imitation of nature.

*United States.*—No. 46. Kingsley, New York. This practitioner has done the profession good service, in producing an excellent imitation of decayed stumps of teeth in porcelain work. Also several cases of irregularities of the second denture. Allen's process upon platina.—This gentleman deserves the thanks of the profession, for the pains taken in producing such exquisite specimens.

48. Fowler & Preterres, exhibit specimens of materials used in the manufacture of mineral teeth, specimens of gold fillings. Allen's process upon platina gilt, finely executed.

No. 50. Ross, New York, American plate teeth, Allen's gum process and some very beautifully executed block work.

*England.*—752. Messrs. Ash & Co., a very fine assortment of tube teeth, exquisitely modeled in shape, they are not excelled by any manufacturer; we wish we could impress this firm of the necessity of a gradation of color, they appear as obstinate as their American competitors, who, as manufacturers, know as much of the niceties and requirements of the operating room, as an Indian coolley. This firm, in the spirit of the times, now manufacture teeth with platina tubes at a very considerable reduction in price; they are in shape and finish quite equal to the gold tube teeth. (We are happy to find *honorable* mention has been made by the "judges" of the exhibition, an instalment certainly for past neglect in these matters.)

762. Harnet, cements for filling teeth, a variety of specimens of mineral teeth, with compressed backs, admirably adapted for short teeth and close bites. Blundell's apparatus for applying cold as an anæsthesia in tooth drawing, manufactured by Messrs. Thornwaite & Co. The apparatus is excellently made, but its anæsthetic effects, when practically applied in operation, does not appear to have realized either the presumed inventor's expectations, (Blundell's,) or the foolish persons who have parted with £100 for the right of patent.

776. Young, *Scotland*, extracting forceps with shifting bits, requiring nine pairs for the extracting of all classes of teeth. Mr. Young, we perceive, has not *patented* his forceps, but if we remember correctly, the invention is not new, having been introduced some years since, at any rate, the mechanical arrangement is very simple, and would no doubt be serviceable to surgeons in the army and navy, who cannot stow away a multiplicity of tooth instruments; they are undoubtedly more ingenious in their construction, more applicable in practice, than a kind of forceps introduced to the profession by Mr. Maclean, at the Medico-Chirurgical Society at London, last winter, on which occasion a gentleman present (Mr. Vassey) remarked, that the forceps referred to, he considered, "improved tooth crushers."

3979. Bidart, gum teeth, and mineral blocks, good colors, but inferior shapes.

*France.*—3980. Messrs. Billard & Son, a large collection of mineral teeth, very inferior in shape and color.

3990. Capron, surgeon's instrument maker, exhibits a collection of badly made tooth forceps and German keys.

3998. Didier, a model of work shop, in which, we presume, some extremely inferior mechanical work exhibited, was manufactured.

4015. Lalemur & Robarts, a very ingenious and simple machine worked by the foot similar to a common lathe, for carving teeth in bone, also in alabaster, some very fine specimens of each were exhibited by an assistant who attended to illustrate the practical working of the machine; in action it is similar to Mr. Jordan's machine used for ornamental carving. Besquet, exhibits some of the finest specimens of carved teeth in the building. Also, imitation of irregularities in the centrals.

4017. Hasler, very inferior specimens of mineral teeth.

4023. Pierret, Brest, a variety of American drills.

4038. Dr. De Villemeur, plates mounted with teeth, and lined with gutta percha; also, a plaster cast representing the loss of the right maxillary arch, which had been supplied by gutta percha mounted with natural teeth. Obturators manufactured from the same materials. A few of Everard's adapted forceps, with circular joints, which the worthy doctor claims as his own invention.

4039. Weile, forceps for extracting teeth perpendicularly; a very clever invention for making spiral springs; also, a contrivance for securing pieces of mechanical work, in cases of large suction pieces.

4059. Cacau, a tolerably well carved piece in hippopotamus; mineral mounted upon platina plates.

10,101. Royer, the most inferior specimens of carved teeth in the whole exhibition.

10,104. Simon, exhibited at the London exhibition, 1851, on the present occasion, his handicraft is upon a *par* with his former attempt; a combination of badly carved bone specimens and old fashioned tooth instruments, representing the key and forceps.

10,105. Soieplet, old fashioned methods of regulating the second denture, with models before and after treatment.

10,110. Wissuer, this exhibitor not only gives us quantity but quality, in the shape of a set of teeth excellently carved in bone, about the size of a modern warming pan, also a smaller specimen equally well executed.

10,072. Gillet, anatomical *carving*, showing the canal and pulp cavities of the teeth. Bone teeth carved, and mineral teeth mounted; the anatomical piece of carving was very beautifully and accurately finished.

10,073. Gion, artificial work upon gold plates and mechanical methods of treating irregularities. Aarboville says, that he has a *patent* for rendering bone indestructible when inserted in the mouth, and exhibits specimens before and after insertion. A *patent* also for making and lining the sockets with mother of pearl; in fact, he appears to be a compound of bone, mother-of-pearl and parchment deeds. Dr. Auroux, exquisite specimens of the teeth of man and horse in wax; a specimen of the new metal aluminium. Desirabode, some very choice pathological specimens of ossific union of the permanent teeth, centrals and molars, exostosis, &c., a case set round with crowns of teeth, perpendicular extracting instruments, teeth mounted upon gold and bone sockets. If publicity be of any service in making a practice, we are certain that Mr. Desirabode must absorb all the patients visiting Paris, for it must cost him a decent fortune for show cases and address cards distributed to passing pedestrians.

